# Use of a Critical Constructivist, Community-Engaged Approach to Understand Commercial Determinants of Breast Cancer: The Situational Scoping Method

**DOI:** 10.3390/ijerph22121873

**Published:** 2025-12-17

**Authors:** Cristin E. Kearns

**Affiliations:** 1 Preventive and Restorative Dental Sciences, School of Dentistry, University of California, San Francisco, CA 94122, USA; cristin.kearns@ucsf.edu; 2 Philip R. Lee Institute for Health Policy Studies, University of California, San Francisco, CA 94158, USA

**Keywords:** commercial determinants of health, qualitative research, industry documents, community-engaged research, breast cancer

## Abstract

In the digital age, online industry documents have become an available and abundant source to inform qualitative health research on the commercial determinants of health (CDOH), including how corporations shape knowledge, policy, and public perception to protect business interests. This paper introduces the situational scoping method, a rigorous and transparent qualitative approach rooted in critical constructivism designed to conduct an overview of large databases of industry documents and systematically map industry responses to external events perceived as threats or opportunities. Developed through a pilot study on environmental exposures and breast cancer, using the UCSF Industry Documents Library, the method consists of three stages: (1) identification of a broad range of external events over time perceived by industries as a threat or opportunity to business interests; (2) selection of a sample of external events for further analysis; and (3) social world/arena mapping of industry responses to selected external events. Conducted by a transdisciplinary team with community partners, the method builds on and enhances traditional tobacco documents and CDOH research by integrating participatory action and collaborative analysis of digital archives. It also offers a transferable framework for examining corporate influence across sectors. This work contributes to emerging public health methodologies that confront commercial power through critical, community-engaged inquiry essential for emancipatory action.

## 1. Introduction

Qualitative health research in the digital age is increasingly turning to secondary sources, such as documentary and digital data, due to their availability and abundance [1]. Over the last 20 years, the University of California, San Francisco Industry Documents Library (IDL) has become an important data source for researchers interested in understanding commercial determinants of health (CDOH) [2]. Launched in 2002, the IDL originally housed millions of documents publicly disclosed in litigation against the tobacco industry in the 1990s. It was one of the earliest digital libraries to provide access to its collections on the internet. IDL research has resulted in over 1100 publications that have shed light on the wide array of tobacco industry practices to promote its products [3]. This research has been instrumental in reducing harm from tobacco products by bolstering tobacco control efforts to implement restrictive regulations, legislation [4,5,6], and a World Health Organization international treaty [7], among other public health efforts. In response to researcher interest in cross-industry practices, the IDL staff began acquiring documents from other industries [2]. As of May 2024, the IDL added 5 new archives. These include the Drug Industry Documents Archive launched in 2006, the Chemical Industry Documents Archive launched in 2017, the Food Industry Documents Archive launched in 2018, the Fossil Fuel Industry Documents Archive launched in 2019, and the Opioid Industry Documents Archive launched in 2022. The IDL now contains more that 140 million pages in over 18 million documents [8]. More than 125 publications based on these new archives have been published since 2008 [3].

The large corpus of material contained within the IDL presents both opportunities and challenges to qualitative health researchers. These archives offer unprecedented access to internal company documents. Such materials reveal tactics to increase product consumption through marketing, public relations, lobbying and other social and political strategies. They also illuminate efforts to weaken regulatory and legislative control of their products; as well as any internal knowledge about a product’s health effects not yet known to the public health community. Challenges include the need for qualitative health researchers to have a strong understanding of IDL search methods [9] and complex data management strategies [2]. Researchers are also required to make decisions about which search terms retrieve the most relevant material, and screening the documents for relevancy can be a time-consuming process [9]. The IDL is also frequently updated as new documents become available. Interpreting the documents can also present a challenge, as analytical practices used in industry documents research have been inconsistent and are often superficially described [10].

Public-health oriented researchers developed tobacco documents research methods, the primary method in use to conduct research on online industry documents, as they sought to make sense of the contents of the tobacco industry documents [4,5,9,11,12,13]. Early work in this field focused heavily on search strategies and chronological documentation, often treating documents as objective evidence. Carter described tobacco document research methods as being initially dominated by a “descriptive mainstream” where researchers acted as passive conduits, offering little theoretical reflection [14]. Over time, researchers began to view documents as socially constructed texts requiring contextual interpretation. However, formal connections to methodology remained rare. Carter called for greater transparency in analytic choices and encouraged the adoption of interpretive approaches such as discourse, case study, or ethnographic analysis to strengthen rigor and depth in document-based research [14].

In 2007, Carter and Little presented a systematic framework for evaluating the quality of qualitative research that transcends standardized reporting checklists and facilitates innovation and diversity in qualitative research practice [10]. This framework demonstrates internal consistency through three foundational concepts: epistemology, methodology, and method. Epistemology refers to a theory of knowledge. Carter and Little argue that every qualitative researcher has a position (often implicit) on how knowledge should be created, which informs methodological choices. Methodology is the logic a researcher uses to produce knowledge and includes the description, explanation, and justification of method selection, as opposed to the method itself. Researchers benefit from guidance from these methodologies to construct their own “logic-in-use” as they develop a strategy to formulate, articulate, analyze, and evaluate their methods.

This paper applies Carter and Little’s framework to develop an innovative qualitative method, the situational scoping method, for application in industry-document research. The situational scoping method was developed for a pilot study to investigate if there are documents in the publicly available UCSF online Industry Documents Library related to breast cancer. This paper reports on (1) the epistemological and methodological influences and foundational methods that inform the situational scoping method; (2) the use of the situational scoping method in the pilot study; and (3) the advantages and challenges of the situational scoping method compared to traditional tobacco documents research methods. By explicitly addressing epistemology, methodology, and method, this approach helps fill critical gaps in how qualitative research engages with CDOH.

Traditional tobacco documents research has revealed the numerous and hidden ways in which the tobacco industry has shaped the public’s knowledge about its products. These strategies have delayed regulation, legislation, and limited public awareness. As a result, communities have faced prolonged and harmful exposure to tobacco products. However, the primary focus on the tobacco industry in public health has led to “tobacco exceptionalism”. This concept refers to the perception that “transnational tobacco corporations [have been] perceived as a pariah industry differentiated by conduct and product characteristics from other commercial actors” [15]. The situational scoping method is distinct from traditional tobacco documents research in that it explicitly articulates an epistemological position which views the tobacco, drug, chemical, food, and fossil fuel industries as powerful, dominant actors. These industries strategically produce, validate, and manage knowledge both proactively and reactively to protect business interests at the expense of the public interest. One objective of the situational scoping method is to increase the public’s awareness of widespread industry construction of knowledge about their products, and the ways in which this knowledge has been adopted without critical reflection.

## 2. Pilot Study Background

In March 2021, the California Breast Cancer Research Program (CBCRP) issued a Request for Proposals (RFP) to support a single project investigating whether documents in the UCSF Industry Documents Library (IDL) address corporate knowledge and strategies related to breast cancer [16]. A preliminary search using the term “breast cancer” returned over 55,000 documents across all collections. Yet, despite the scale of this material, none of the 800+ peer-reviewed publications based on the IDL had focused directly on breast cancer. The RFP highlighted longstanding concerns about environmental exposures—such as secondhand smoke, petrochemical byproducts, chemicals, and pharmaceuticals. It called for exploratory research into whether internal industry communications in the IDL could illuminate how companies responded to growing evidence linking their products to breast cancer. This gap represented an important opportunity to advance research on the CDOH in the context of a leading women’s health issue.

The RFP specified that applicants should apply established tobacco documents research methods and include at least one team member with expertise in this area [16]. Advocacy involvement was a central requirement, consistent with CBCRP’s broader commitment to participatory research and health equity. Advocates were expected to help shape research questions, goals, and ensure community relevance. The RFP also encouraged collaboration with community partners knowledgeable about industry tactics, scientific manipulation, and environmental contributors to breast cancer. This approach is well-suited to examining the commercial forces that shape public health outcomes. Our team partnered with Breast Cancer Action (BCAction), a feminist grassroots advocacy organization with a long history of exposing corporate hypocrisy in breast cancer campaigns and environmental health [17]. Together, we were awarded the CBCRP contract in July 2021.

The objective of the pilot study was to determine whether there is data in the UCSF IDL about the tobacco, chemical, pharmaceutical, food, and fossil fuel industries that could contribute to finding innovative solutions to reduce suffering from breast cancer and move science closer to eliminating the disease. We hypothesized that the IDL contained data that would demonstrate that the tobacco, chemical, pharmaceutical, food, and fossil fuel industries have worked to suppress public awareness of the link between environmental exposures and breast cancer. Our aims were to: (1) describe internal research sponsored by the tobacco, chemical, pharmaceutical, food, and fossil fuel industries related to environmental exposures and breast cancer that was not publicly released; and (2) describe public relations campaigns sponsored by the tobacco, chemical, pharmaceutical, food, and fossil fuel industries designed to influence public opinion about environmental exposures and breast cancer.

## 3. The Situational Scoping Method

The situational scoping method is designed to investigate how industries respond over time to external events perceived as threats or opportunities to their business interests. “Situational” draws from definition of a situation as “a somewhat enduring arrangement of relations among many different kinds and categories of elements that has its own ecology” which “usually includes a number of events over at least a short period of time, and can endure considerably longer” [18]. Specifically, for the situational scoping method, the situations of inquiry are industry responses over time to external events considered to be threats or opportunities to business interests. Scrutinizing these industry responses to external events follows corporations and organizations as they seek to stay competitive amongst changing social, economic, and political circumstances. I also enables a critical analysis of the contexts and mechanisms through which they do so, utilizing industry documents as data sources within the situational scoping method. “Scoping” refers to the goals of a traditional scoping review, which is designed to apply rigorous and transparent methods to conduct an overview or map a body of literature relevant to research questions.

In this method, university researchers collaborate with community partners to scope industry documents related to external events. These events are examined as potential threats or opportunities to business interests pertaining to a jointly developed research question/s. Examples of external events include emerging research, increased media attention, and new regulation or legislation related to an industry’s product that has the potential to boost or restrict sales.

The Situational Scoping Method stages are:

1. Identification of a broad range of external events over time perceived by industries as a threat or opportunity to business interests.
2. Selection of a sample of external events for further analysis.
3. Social worlds/arenas mapping of industry responses to selected external events.

**Figure 1:**
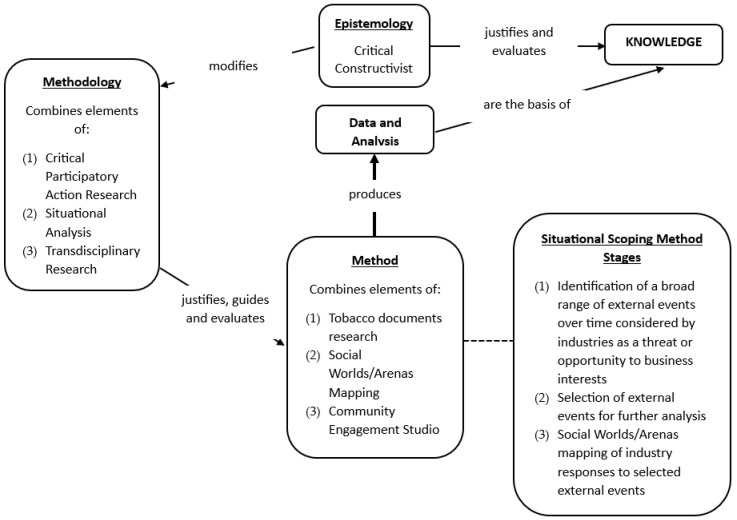
The Relationship Between Epistemology, Methodology, and Method and the Stages of the Situational Scoping Method for Industry Documents Research. Reproduced from Tobacco Control, Han et al., 23 April 2025, Supplementary Material page 1, 2025 with permission from BMJ Publishing Group Ltd. [19].

Figure 1 illustrates the relationship between the Situational Scoping Method and its stages, along with the epistemology, methodology, and methods that underpin it, as described in the following sections.

### 3.1. Epistemological Influences

#### Critical Constructivism

The epistemological position of the situational scoping method is critical constructivism as outlined in Kincheloe’s Critical Constructivism Primer [20]. Critical constructivism draws from a constructivist epistemology, which views the world as a human, or social construction [21]. In a socially constructed world, all forms of perception are shaped by our individual consciousness, meaning that there are no neutral or truly objective perspectives. Knowledge of the world is interpreted by the human agents within the world who create meaning that is shaped by their diverse historical and contemporary experiences and ways of seeing.

Critical constructivists add a critical theory perspective to social constructivism by promoting reflection on the “production of self” [21]. Critical theory posits that many of us are unable to discern the ways in which our social environments have shaped our perceptions of the world, but also our self-concept. An objective of critical theory is to increase our awareness of the ways that dominant power wielders operate to strategically produce, validate, and manage knowledge and our uptake and assimilation of it without us realizing it. Critical constructivists produce a “more complex understanding of the social, political, economic, cultural, psychological and pedagogical world” that links knowledge construction to the privileging of some and the marginalization of others [21].

This epistemological stance supports inquiry into how dominant institutions shape not only public knowledge. It also allows us to examine the conditions under which certain ideas become accepted as truth. These processes often serve commercial interests while marginalizing alternative perspectives.

### 3.2. Methodologies Employed

The situational scoping method combines elements of three methodologies. First, it draws on critical participatory action research, which incorporates impacted community members. Second, it integrates Clarke’s Situational Analysis, which provides analytical tools designed to understand a situation of interest. Third, it adopts a transdisciplinary research orientation, which engages multiple researchers from varied disciplines. The following sections outline how these three methodological traditions inform the situational scoping method and connect to its critical constructivist foundation.

#### 3.2.1. Critical Participatory Action Research

Critical participatory action research (CPAR) is a form of action research that focuses on questions of power and injustice [22]. It involves comprehensive participation by a collective of researchers which includes members of communities most impacted by the issue being studied. Ideally, the research collective engages in all aspects of the research project, including theorizing, creating research questions, selecting methods, conducting analysis, writing up findings, and other dissemination activities. Research methods can be quantitative, qualitative, mixed methods and may be historical and visual or creative and traditional. Regardless of approach, all methods are conducted from the perspective of democratic knowledge production that centers community perspectives. CPAR engages in “collective reflexivity” to examine perspectives grounded in dominant power and oppression while recognizing the biographies and experiences that inform individual and collective perspectives of the researcher collective. Research is linked to action with the intent to reeducate the public, provoke action, and advance social change. Forms of action can include scholarship, social policy, teaching, legal reform, organizing, theater, art, music, and many others.

CPAR aligns with a critical constructivist epistemology as researchers work alongside those who are excluded and subjugated to expose the existing social order as oppressive and unethical. They “ask what are the forces that construct the consciousness, the ways of seeing of the actors who live in it” [21]? These questions are deemed essential for emancipatory action. Understanding how knowledge is used to perpetuate oppression, then becomes the basis for developing strategies to overcome such oppression [21]. The situational scoping method involves a collective of researchers including members of communities most impacted by the issue being studied. When scoping the UCSF IDL documents, the situations of interest refer to external events perceived as threatening by the industries under study related to the project’s research questions. These situations vary across project team members. Each participant draws on their unique knowledge, experience, and opinions about which situations are deemed essential for emancipatory action.

#### 3.2.2. Situational Analysis

Situational analysis is a methodology developed by Clarke that provides a set of analytical tools and exercises designed to understand a “situation of interest” [23]. Clarke explicates the theoretical grounds of situational analysis, which has evolved over time, in a full chapter in *Situational Analysis* [18]. She draws inspiration from Straussian grounded theory, symbolic interactionism, American pragmatism, Chicago School conceptions of social ecologies, as well as aspects of post-structural and postmodern theoretical work from Foucault (discourse, disciplining, power/knowledge, dispositif), Deleuze and Guattari (rhizomes and assemblages), Latour, Callon, Law, and Akrich (actor–network theory, nonhuman actants) and Haraway (human/nonhuman cyborgs, situated knowledges). However, the theoretical backbone of situational analysis is Strauss’ social worlds/arenas theory [18].

Clarke describes social worlds as “groups of varying sizes that generate a life of their own; for example, a recreation group, an occupation, a theoretical tradition, or even a discipline” [18]. Social worlds, according to Clarke, create “shared perspectives that form the basis for both individual and collective identities and for commitment to collective action” and “are also universes of discourse” that are “situation-dependent” [18]. Multiple social worlds that focus on various issues, particularly those that are “debated, negotiated, fought out, forced and manipulated by representatives” constitute an arena of concern [18]. Social worlds and arenas, then are sites of negotiation, and SA’s empirical questions, then, focus on “who cares” about the various issues, and “what do they want to do about it” [18]?

Situational analysis aligns with a critical constructivist epistemology. It permits “analyses of an array of collective human social entities and their actions, discourses, and power relations in the situation of concern” [18]. Situational analysis also supports analysis of historical discourse materials, particularly related to “studies of long-term controversies” [18] such as Garrety’s analysis of the “cholesterol controversy” in public health [24]. Situational analysis aligns with document scoping, in that it “can be used as an analytic exercise to get researchers moving into and then around the data.” [23]. Data collected with the situational scoping method can be analyzed using situational analysis’ analytical tools. The visual mapping tools are also useful for sharing in collaborative research environments.

#### 3.2.3. Transdisciplinary Orientation

A transdisciplinary orientation in research emerged in response to common mismatches between knowledge needed to solve societal problems and knowledge produced in siloed academic settings that can be limited by over-specialization, fuzzy disciplinary boundaries, and compartmentalized scientific knowledge [25]. Transdisciplinary research aims to respond to societal requests for knowledge in a way that breaks academic silos and engages in mutual learning with members of society. Conducting transdisciplinary research requires bridge-building between different academical cultures, such as between the social sciences and natural sciences, as well as engaging in mutual learning by stepping into the problem field. The situational scoping method utilizes a transdisciplinary research team to collaborate in the analysis and contextualize a large amount of textual material to enhance analysis and reduce the amount of time needed for scoping.

### 3.3. Methods Selected

The situational scoping method combines and expands on elements of three methods including tobacco documents research, social worlds/arenas mapping, and a community engagement studio. These elements are the practical activities of research that describe sampling, data collection, management and analysis, and reporting [10].

#### 3.3.1. Tobacco Documents Research

Tobacco documents research refers to procedures developed by public health researchers to search and analyze internal tobacco industry documents [14]. Search procedures have evolved since the first public release of documents in 1994 with technological advances such as document availability on the world wide web, optical character recognition, and online document storage, organization and annotating capabilities [9]. Tobacco documents research search strategies employ a snowball technique, beginning with a list of search terms generated from research questions. Additional search terms are identified iteratively by analyzing metadata and document content. These terms might include key individuals, organizations, products, project names, and budgets, among others. When large numbers of documents are returned, researchers review the first 50 to 350 documents. Documents can be sorted by ‘relevancy’, which ranks them based on the ratio of search terms to the number of pages in the documents. Alternatively, they can be sorted by date, to help construct a timeline of events and important time periods. Documents are excluded if they are duplicates or found not to be relevant to the research question. Included documents are typically analyzed through informal hermeneutic thematic analysis, not tied to a particular qualitative research tradition [14]. Additional data sources, through a process of triangulation, may be identified to contextualize and deepen understanding of topics. Search strategies are documented, and research memos written to aid in analysis.

Similarly to traditional tobacco documents research, the situational scoping method utilizes document search technology, including optical character recognition, and online document storage, organization, and annotating capabilities. It also employs snowball-style search based on iterative development of search terms; Additionally, the method constructs a timeline of events and important time periods [2,9]. In contrast, the situational scoping method focuses on providing a high-level overview of the provenance, frequency, and distribution of documents containing search terms (e.g., by decade, by product type, document type). The method also involves close collaboration with transdisciplinary researchers and community partners to identify iterative search terms. Document review is intentionally limited to “high-value” documents (e.g., internal memos and reports, formerly confidential and privileged documents). Finally, the method organizes analyses around situations of interest identified as relevant by the entire project team. These are supported by semi-structured memos, and visual mapping tools.

#### 3.3.2. Social Worlds/Arenas Mapping

**Figure 2:**
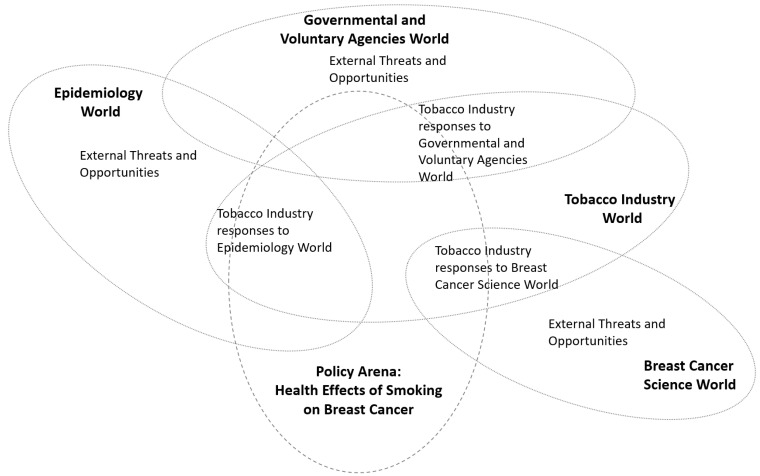
An exemplar abstract social words/arenas map used in the situational scoping method. The arena represents a policy arena in which the health effects of smoking on breast cancer are debated. Social worlds represent groupings of actors and organizations producing external threats or opportunities to the tobacco industry’s business interests, with the tobacco industry social world representing industry response to the threats and opportunities.

Clarke developed an iterative social worlds/arenas mapping method, supported by a set of empirical questions and structured memos, to assist researchers in understanding situations of interest [26]. “Social worlds/arenas maps are usually first done one you have gathered some data…to make collective sense out of it” [18]. A “social world” refers to a group of any size that is connected through shared perspectives, discourses, identities, and collective action. An “arena” forms when representatives of multiple social worlds debate, negotiate, fight out, force or manipulate various contested issues. The situational scoping method utilizes social worlds/arenas mapping to visualize industry responses to external events over time perceived by IDL industries as a threat or opportunity to business interests supported by semi-structured memos developed by Clarke [18]. Figure 2 contains an abstract social worlds/arenas map that is representative of the mapping work conducted in the situational scoping method.

#### 3.3.3. Community Engagement Studio

The Community Engagement Studio (CE Studio) approach was developed by the Meharry–Vanderbilt Community Engaged Research Core in 2009 to more effectively engage communities in research [27,28]. A CE Studio is a “structured program [that] facilitates project-specific input from community and patient stakeholders to enhance research design, implementation, and dissemination.” [27]. Community stakeholders serve in a consultative role and are compensated for their time. CE Studios consist of two-hour face-to-face meetings facilitated by an experienced, neutral moderator who works to ensure equitable sharing. They begin with brief presentations from the researcher, followed by two or three questions presented to a stakeholder panel, and a moderated discussion. The research team gives a brief presentation about the project and poses specific questions to community experts. A facilitator then guides the discussion that follows to elicit authentic and constructive feedback. Advance preparation by the research team and community experts is essential. The situational scoping method incorporates the CE Studio approach into project team meetings to guide collaboration, and to gather feedback on document search terms, situations of interest and social worlds/arenas maps.

## 4. Situational Scoping Method Procedures in the Pilot Study

The situational scoping method was operationalized through a 12-month pilot study funded by the California Breast Cancer Research Program and conducted in partnership with Breast Cancer Action (BCAction). Table 1 outlines the procedures used to implement the method, organized by project team (all project participants) and research team (academic researchers) activities. During Stage 1, the team identified a wide range of external events perceived by industry actors as threats or opportunities; these are listed in Table 2. In Stage 2, ten topic areas were selected for further analysis. Table 3 summarizes focused document searches conducted for each topic. Figure 2 presents an abstract example of a social worlds/arenas map constructed during Stage 3. Throughout the project, the research team engaged in explicit reflexive practices to address positionality and power dynamics inherent in collaborative, community-engaged research. The Community Engagement Studios were intentionally facilitated by a neutral moderator who structured the discussions to ensure equitable participation and to mitigate academic hierarchies, disciplinary authority, and institutional power. Team members were encouraged to articulate their assumptions, prior experiences with industry influence, and potential blind spots. This reflexive orientation shaped decisions about search terms, interpretations of documents, and selection of external events, ensuring that analytic priorities were not solely driven by academic researchers but were co-constructed with community expertise.

## 5. Summary of Results

The situational scoping method produced three primary outcomes across the pilot study. First, the project team identified a broad set of 53 external events across tobacco, food, chemical, pharmaceutical, and fossil fuel industry archives that were perceived by these industries as potential threats or opportunities related to breast cancer. These external events ranged from emerging scientific studies, regulatory actions, and shifts in public discourse to internal industry assessments of breast cancer research trends. Table 2 presents these events by industry and topic area.

Second, through collaborative review in the Community Engagement Studios, the project team narrowed these 53 external events to ten focal topics for in-depth document analysis. These topics reflected areas of the highest relevance to the research questions and included: (1) Tobacco industry-funded research about breast cancer (1970s–1980s); (2) Tobacco industry-funded research about breast cancer (1990s); (3) 1997 California Environmental Protection Agency Report: Health Effects of Exposure to Environmental Tobacco Smoke; (4) 2005 California Environmental Protection Agency Report: Health Effects of Exposure to Environmental Tobacco Smoke: (5) Association of Hormonal Replacement Therapy with Breast Cancer; (6) Glyphosate and Breast Cancer; (7) Benzene and Breast Cancer; (8) Dichlorodiphenyltrichloroethane (DDT) and Breast Cancer; (9) Recombinant Bovine Somatotropin and Breast Cancer; (10) Dietary Fat and Breast Cancer.

Third, researchers constructed preliminary and revised social worlds/arenas maps for each of the ten focal topics. These maps depicted the relationships among key actors, organizations, discourses, and industry strategies across time. Using Clarke’s mapping tools, each map documented how industries monitored external events, framed scientific debates, sponsored or interpreted research, and designed public relations responses. Preliminary findings from this pilot study are being further developed in subsequent publications. For example, Han et al. have published an in-depth case study of tobacco industry–funded research on breast cancer using this methodological framework and early results [19].

## 6. Discussion

### 6.1. Potential Advantages of the Situational Scoping Method

The situational scoping method enhances traditional tobacco documents research in several ways. First, knowledge is produced through comprehensive participation throughout all aspects of the research by a collective of researchers and members of communities most impacted by the issue being studied. Second, the situational scoping method defines a systematic approach to assess a large corpus of industry documents and procedures to identify the most relevant material to support the goals of action research, such as re-educating the public and advancing social change related to issues of power and injustice. Third, decision-making and data collection and analysis activities, supported by visual tools, occur in a collaborative environment in which transdisciplinary researchers and community members can provide contextualization and triangulation data in real time. This also creates an environment in which a less experienced documents researcher can be mentored by more experienced researchers. Fourth, the situational scoping method can be applied to any industry data source comprising digital documentary data. And finally, the situational scoping method can accommodate multiple definitions of an impacted community which could range from individuals directly exposed to harmful products, to opinion leaders (e.g., policymakers, health professionals, voluntary organizations) with decision-making power over policies or scientists conducting safety and efficacy research, who have been the targets of industry influence attempts. From a critical constructivist perspective, any community member or organization that have unknowingly adopted knowledge constructed by industries producing a harmful product could benefit by participating in a project using the situational scoping method that increases their awareness of industry influence attempts.

The situational scoping method also provided a strong foundation for dissemination activities that aligned with our critical constructivist epistemology, particularly its emphasis on emancipatory outcomes and the deconstruction of power. Building on our preliminary findings, BC Action conducted focus groups with ten advocacy organizations to assess how the emerging results could support their efforts to confront industry influence. These discussions revealed substantial variation in advocates’ awareness of cross-industry tactics, particularly regarding food and pharmaceutical companies, and illuminated how industry-funded front groups and misleading scientific narratives shaped organizational decision-making. Participants identified immediate applications of the findings, including avoiding inadvertent partnerships with industry-aligned entities, strengthening guidance on funding and sponsorship decisions, and developing fact sheets, webinars, and training materials to help communities recognize industry tactics. This work culminated in a tailored breast cancer–focused track within the annual UCSF Industry Documents Workshop, where advocates received hands-on training in document searching, analysis, and strategy identification, supported by archivists and faculty mentors. Collectively, these dissemination efforts redistributed analytic capacity to communities, expanded advocates’ ability to independently interrogate corporate influence, and translated the situational scoping method’s critical commitments into concrete emancipatory practice.

Another advantage of the situational scoping method is that is aligned with situational analysis. The findings produced with the situational scoping method provide an overview of the range of documentary material relevant to research questions that are contained within the documents databases. It uses social worlds/arenas analysis, which is only one of three cartographic approaches available in situational analysis “to understand the dense complexities of a particular situation.” [32]. Future projects can build on the findings generated from projects using the situational scoping method to conduct in-depth analysis of documents, as well as through the incorporation of in-depth interviews, ethnographic observations, or other textual sources such as websites and social media. Situational analysis “allows researchers to draw together studies of discourse and agency, action and structure, image, text and context, history and the present moment—to analyze complex situations of inquiry” [18].

The situational scoping method also aligns with broader methodological developments in qualitative and mixed-methods health research. Recent work in digital ethnography and multi-sited digital inquiry has emphasized the need for analytic approaches capable of systematically engaging large, heterogeneous digital corpora while retaining interpretive depth [33,34]. The situational scoping method contributes to this expanding methodological terrain by offering a structured, participatory, and theoretically grounded process for navigating large digital archives. Its integration of community expertise, collaborative mapping, and iterative memoing complements emerging mixed-methods approaches that combine documentary analysis with interviews, policy analysis, and computational tools.

The situational scoping method is especially timely given the expansion of the UCSF Industry Documents Library to include internal records from industries beyond tobacco, including pharmaceutical, food, fossil fuel, and opioid sectors. These collections provide a critical opportunity to advance the emerging field of CDOH, which seeks to understand how corporate actors shape health through marketing, political influence, and the construction of knowledge. As articulated in recent foundational work on the CDOH field, new methods are needed to interrogate corporate practices across sectors with rigor and reflexivity [32]. The situational scoping method offers a transferable framework to meet this need by combining collaborative search, visual mapping, and community engagement to expose how knowledge is produced and managed in service of commercial interests. As access to internal corporate documents continues to grow, this method supports the development of CDoH research by identifying cross-industry strategies, revealing structural mechanisms of influence, and generating findings that are meaningful for both academic and advocacy-based interventions. The breadth of peer-reviewed studies listed in the UCSF IDL bibliography, which spans multiple industries, topics, and geographies, illustrates that researchers are increasingly recognizing the value of internal-industry documents across sectors [3].

### 6.2. Challenges in Using the Situational Scoping Method

The situational scoping method requires an investment in training related to document search methods, methodologies and methods, and community engagement activities. Community participants can benefit from participating in document search training by gaining additional insight into the research process, however their capacity and motivation to do so may vary. Transdisciplinary researchers with a range of expertise valuable to the project may have limited training in qualitative research traditions, necessitating education and training related to epistemology, methodology, and methods. Researchers may also have varied experience with community engaged research and may require education and training about appropriate community engagement practices.

The situational scoping method also requires flexibility with analytical tools. Social worlds/arenas mapping can be carried out with a range of available applications, and the maps can be constructed in a number of different ways. Initial map construction often varies by researcher and may require multiple iterations and input from the project team. Additionally, our team experimented with combining other elements from the cartographic tools that are part of situational analysis. For example, discourses, which are typically part of positional maps, were included in social worlds/arenas maps.

Finally, the amount of time needed to convene the project team via the community engagement studios can vary depending on the amount of documentary material being scoped. It can be challenging to identify the amount of material that is realistic for project team members to evaluate ahead of time, as well as the amount of time that is needed or available for the project team to convene. Flexibility is needed to adapt these sessions to best serve the team members and project goals.

### 6.3. Added Value of the Situational Scoping Method

While traditional document analysis, tobacco documents research, and situational analysis each provide important tools for interrogating corporate influence, the situational scoping method offers several distinctive contributions. First, existing approaches provide limited guidance for systematically scoping very large, multi-industry digital archives like the UCSF Industry Documents Library. By integrating structured search procedures, collaborative memoing, and iterative mapping at the scoping stage, the situational scoping method provides a transparent and auditable process for narrowing a very large document corpus into a manageable set of high-value materials for further analysis.

Second, traditional tobacco documents research emphasizes sequential snowball searching and in-depth document interpretation, but it does not typically incorporate multiple disciplinary perspectives or community expertise in real time. In contrast, the situational scoping method embeds transdisciplinary collaboration and community engagement throughout the process, which complemented individual searches and supported collective identification and contextualization of external events and industry response strategies.

Third, although situational analysis has been applied to historical documents, including analyses of long-term scientific and policy controversies, it does not offer procedures for identifying, narrowing, or selecting documents within vast digital archives. The situational scoping method was developed specifically to address the methodological challenges posed by multi-million–document collections like the Industry Documents Library. It integrates search strategy development, collaborative screening, iterative memoing, and preliminary mapping to guide decisions about which situations, events, and documents warrant deeper analysis.

Together, these innovations enable a more systematic, participatory, and reflexive approach to scoping digital industry archives. The method does not replace traditional document analysis or situational analysis; rather, it strengthens these approaches by providing a rigorous and transparent foundation for subsequent analytic work.

## 7. Conclusions

The situational scoping method was developed as a qualitative tool for scoping digital industry documents databases, such as the UCSF Industry Documents Library, to conduct an overview of industry responses over time to external opportunities or constraints related to business interests. Rooted in a critical constructivist epistemology, the situational scoping method directly acknowledges issues of power and injustice and the unknowing adoption of industry-constructed knowledge among various communities. The situational scoping method is a form of critical participatory action research that engages impacted communities, together with a transdisciplinary research team, to increase the public’s awareness of industry activities that negatively impact health and to inform action strategies.

## Figures and Tables

**Table 1:** Situational Scoping Method Procedures Used in Breast Cancer Pilot Study. Stage 1 was initiated in August 2021, and Stage 3 completed in July 2022.

Project Team Procedures	Description	Research Team Procedures	Description
Team Formation	The project was led by a principal investigator (CK) in collaboration with Breast Cancer Action (BCAction). Initial contact was established after both parties attended a CBCRP informational session in February 2021. Two exploratory meetings were held to discuss a potential collaboration. BCAction brought experience working with a UCSF research team and was familiar with industry tactics, manipulation of science, and environmental contributors to breast cancer, though with limited prior experience using the IDL. The organization agreed to partner on the CBCRP grant application in March 2021. The project was also supported by two UCSF IDL archivists and a facilitator with expertise in participatory action research.	Team Formation	A transdisciplinary research team was assembled, comprising 11 investigators with expertise in industry documents research, tobacco, food, and chemical industry studies, public health, communications, medical anthropology, sociology, political science, environmental health, and breast cancer epidemiology. The team included participants at all career stages, from master’s students to senior faculty. Supplemental funding was provided by the UCSF Center for Tobacco Control and Education and the UCSF Environmental Research and Translation for Health Center to support two postdoctoral scholars. Additional support for two master’s students came from the University of Nevada, Reno.
Grant Submission	The PI, BCAction, and the facilitator collaborated through email and Zoom meetings to co-develop grant deliverables, including the lay abstract, BCAction’s letter of commitment, advocacy involvement plan, program responsiveness narrative, specific aims, milestones, and the research plan. The proposal was funded in June 2021, with a start date of 1 August 2021, and a 12-month project period.		
Stage 1: Identification of a broad range of external events perceived by IDL industries as a threat or opportunity to business interests			
(1) UCSF IDL Documents Training	Training needs for this project included educating project team members unfamiliar with the IDL on the public health impact of industry documents work and instruction on how to search the digital collections. IDL archivists conducted an initial IDL training session for BCAction and research team members who would be searching the documents in August. Training content included: overview of the IDL (how documents are obtained and ingested, how to search one industry vs. one collection at a time, understanding duplicate documents, searching metadata, and features of an individual IDL account); IDL search features and tools (browse, date ranges, facets, document types including restricted, formerly confidential, court case, area/box/folder, mentioned); and search demonstrations. A follow-up training was held in December on advanced search strategies including the snowball method, building, and revising search queries, advanced search tips—wildcard/fuzzy search, proximity search.	(2) Assigning the IDL Collections	After the preliminary search of the entire IDL for the search term “breast cancer” returned 55 K documents, the research team met to decide how to assign analysis of the documents to individual members. Four researchers were assigned to the tobacco industry collections. One researcher was assigned to the food industry collections, and one researcher was assigned to the chemical, drug, and fossil fuel industry collections (combined).
(4) Community Engagement Studio #1	The first CE Studio meeting was held in late August. The PI, BCAction and facilitator met prior by Zoom and communicated subsequently by email to plan the first project meeting and co-create the agenda. Project team members received an agenda ahead of time to prepare them to give input. At the CE Studio, an overview of the grant and key deliverables were reviewed. The facilitator led a roles and partnership discussion. Team members were asked to share thoughts on what they uniquely brought to the collaboration and what they were hoping to learn. Successes and challenges of partnerships were discussed, including learnings from past experiences. Partnership principles were developed. The research team shared preliminary findings on collection and document characteristics and received feedback from the larger project team on the relevance of initial themes, organizations, and actors to the research questions. Additional relevant search terms were suggested.	(3) IDL Collection Characteristics	During August, research team members collected data on the number of collections containing documents returned with the search term “breast cancer.” collection characteristics (type, provenance) frequency and distribution of documents (e.g., by decade, by product type, document type). For example, the largest number of tobacco and food industry documents returned were produced between the 1970s and 1990s, most drug industry documents were produced in the 1990s and 2000s, most chemical industry documents were produced in the 2000s, and most fossil fuel industry documents were produced in the 1990s. Initial impressions on general themes of document content were recorded, including important time periods, tobacco brands, foods, chemicals and organizations of interest. Four ten-minute presentations were created in preparation to share this information at the first CE Studio.
		(5) Identifying External Events of Interest	Researchers sorted documents returned with the search term “breast cancer” by decade and were instructed to identify “high value” IDL documents, such as internal memos and reports, formerly confidential and privileged documents. Document abstracts were screened for relevance to the project’s research questions before reading a document in-depth. Close attention was paid to industry surveillance of the policymaking environment, the news media and the scientific media for external events related to breast cancer perceived by the industries as threatening, as well as the research, public relations, marketing, and advertising activities planned in response. Situations of interest were tobacco, food, drug, chemical, and fossil fuel industry responses to external events over time related to breast cancer perceived by these industries as threats or opportunities to demand for their products. Using Padlet, a cloud-based real-time collaborative web platform in which users can upload, organize, and share content to virtual bulletin boards [29], researchers recorded potential situations of interests, including various elements and actors, together with links to high-value documents. Researchers had flexibility with how Padlet boards were constructed, which was influenced by archive size and number of collections with relevant documents. Investigators incorporated research memos into the mapping process by moving between document searches, creating, and editing situational maps, and writing research memos to aid in making conceptual and relational connections. Memos captured document keyword search strategies and documents retrieved and reviewed. The research team met weekly to review progress and received input from other research team members on searches, findings, contextualization, and advice to avoid “analysis paralysis.” At the end of the scoping and initial assessment phase, researchers were asked to construct one Padlet Board documenting the identified situations of interest, key actors, and industry discourses. Fifty-three breast cancer-related external events were identified across the 5 IDL collections ranging from the 1969 U.S. Department of Health, Education and Welfare Commission on Pesticides and their Relationship to Environmental Health to the 2017 Environmental Protection Agency Cancer Assessment Review Committee’s review of glyphosate toxicity (Table 2). Events were grouped by industry into topics: tobacco industry archives—genetic causation, smoking, environmental tobacco smoke, smoking and reproductive health, breast cancer prevalence, aspirin use, and dietary explanations for breast cancer; chemical and fossil fuel industry archives—glyphosate, benzene; drug industry archives-Premarin; food industry archives—chemicals/pesticides in foods, dietary fat, alcohol, synthetic estrogen, growth hormones, obesity and physical activity.
Stage 2: Selection of external events for further analysis			
(6) Community Engagement Studio #2	The second quarterly CE Studio was held in November. After a brief overview of the 2nd phase of the project, research team members presented the results of their initial scoping and assessment work, including the Padlet Boards documenting potential situations of interest. A facilitated discussion was conducted to elicit feedback on events/situations most likely to reveal data related to our research questions was elicited from the project team. Ten topic areas were selected for further analysis: (1) Tobacco industry-funded research about breast cancer (1970s–1980s); (2) Tobacco industry-funded research about breast cancer (1990s); (3) 1997 California Environmental Protection Agency Report: Health Effects of Exposure to Environmental Tobacco Smoke; (4) 2005 California Environmental Protection Agency Report: Health Effects of Exposure to Environmental Tobacco Smoke: (5) Association of Hormonal Replacement Therapy with Breast Cancer; (6) Glyphosate and Breast Cancer; (7) Benzene and Breast Cancer; (8) Dichlorodiphenyltrichloroethane (DDT) and Breast Cancer; (9) Recombinant Bovine Somatotropin and Breast Cancer; (10) Dietary Fat and Breast Cancer.	(7) Additional Document Searches	The research team conducted additional document searches to support an in-depth review of each topic (Table 3). We found that the IDL contains at least 13,186 documents indicating that the tobacco industry’s Council for Tobacco Research (CTR) sponsored research projects related to breast cancer between the 1970s–1990s; 1067 documents related to Philip Morris’ and RJ Reynolds’ responses to events leading up to the 1997 and 2005 California Environmental Protection Agency Report on environmental tobacco smoke (ETS); at least 175 documents related to Wyeth-Ayerst’s communication campaign developed in response to emerging of evidence in 2002 that their hormonal replacement therapy (HRT) drug Premarin increased breast cancer risk, and that HRT was not beneficial to women with or at increased risk for coronary heart disease; at least 80 documents related to Monsanto’s response to the 2015 International Agency for Research on Cancer’s systematic review of the carcinogenic potential of glyphosate which concluded it is a probable carcinogen; at least 212 documents related to Shell Oil and other oil industry actors’ (e.g., American Petroleum Institute) hiring of the consultant group Environ Corporation to re-analyze data reviewed in 1984 by the California Department of Health Services to cast doubt on the value of animal models to understanding benzene exposure risk in humans-including mouse models which demonstrated ovarian and mammary gland tumors; 102 documents related to DDT and breast cancer; 143 documents related to recombinant bovine somatotropin, breast cancer, and Monsanto’s relationship with the Harvard University Department of Nutrition in 1980s/90s; and over 10,000 documents related to dietary fat and breast cancer.
Stage 3: Social worlds/arenas mapping of industry responses to selected external events			
		(8) Social worlds/arenas mapping and memoing training	CK provided training to the research team on identifying situations of interest; creating social worlds/arenas maps; and on memoing. SA readings were provided on social words/arenas including exemplar projects [18,26,30]. The research team received guidance on drafting a final project memo, including a memo template. Memos included documentation of search strategy, summaries of collection and document characteristics, scoping and initial assessment processes, in-depth review processes, and a summary of key findings, contributions of the research to the specific aims, and implications of the findings to breast cancer prevention, treatment, and policy.
		(9) Preliminary social worlds/arenas mapping	The research team began constructing social worlds/arenas maps using Google Jamboard [31] for each of the ten topics selected for further analysis. Analytic “close-ups” were created for each on social worlds/arena maps with an intense focus on identifying research related to links between environmental exposures and breast cancer and public relations activities designed to influence public opinion related to environmental exposures and breast cancer [18]. The research team met weekly to review progress and received input from other research team members. At the end of the in-depth review phase, researchers were asked to prepare brief presentations on their social worlds arenas maps.
(10) Community Engagement Studio #3	The third quarterly CE Studio was held in February. After an overview of the 3rd phase of the project, research team members presented the results of their in-depth review of documents, including preliminary social worlds/arenas maps. A facilitated discussion was conducted to elicit feedback on content and suggested revisions of the social worlds/arenas maps, as well as key material to include in the final project memos.		
		(11) Iterative social worlds/arenas mapping	Social worlds/arenas maps were finalized and compiled with the final project memos by the research team into one pdf and made available to the entire project team. The research team created final presentations of results.
(12) Advocacy Focus Groups	To inform efforts to disseminate project findings, BCAction conducted 3 focus groups with partners and key stakeholders in June 2022. Focus groups were organized by topic: (1) tobacco industry findings (2) food industry findings, and (2) drug and chemical industry findings and lasted one hour. After introductions, participants were shown a 10 min pre-recorded presentation delivered by members of the research team. BCAction then asked participants 11 questions to elicit feedback on participants prior knowledge of the subject matter, relevance of the findings to participants, ideas for dissemination, and use potential of the IDL for future advocacy work.		
(13) Community Engagement Studio #4	The final CE Studio was held in May. After a brief overview of the final phase of the project, BCAction presented focus group results and research team members presented a high-level summary of their final social worlds/arenas maps and memos. A facilitated discussion was conducted to elicit feedback related to dissemination planning and manuscript preparation. Findings were used to apply for a CBCRP dissemination supplement, which we received to support two symposiums held in 2023.		

**Table 2:** Breast-cancer related external events that were threats or opportunities to industry ^1^.

Year	External Events
Archive: Tobacco Industry Documents	
Topic: Genetic Explanations for Breast Cancer	
1970s	Hereditary cancer research
Topic: Smoking and Breast Cancer (Positive and Negative Associations)	
1977	Potential Association Between Smoking and Breast Cancer: Effects of X-Rays
1979–1983	Negative Association Between Smoking and Breast Cancer (Opportunity for Industry)
1980s–1990s	Studies Linking smoking and breast cancer
1986	Surgeon General Reports
1988	California Proposition 99
1995	Evidence of Increased Risk for Breast Cancer for Long-Time Smokers
1996	Evidence of Weak Association Between Active and Passive Smoking and Breast Cancer
1999	Evidence of Modest Inverse Relationship with Current Smokers and Breast Cancer (Not Past Smokers)
1999	Evidence of Real Association for Passive and Active Smokers and Breast Cancer
Topic: Secondhand/Environment Tobacco Smoke and Breast Cancer	
1980s	Studies about secondhand smoking and cancer
1990, 1993	California EPA’s guidelines on indoor smoking policy and designation of secondhand smoking as carcinogen
1997	California Environmental Protection Agency Report: Health Effects of Exposure to Environmental Tobacco Smoke
2005	California Environmental Protection Agency Report: Health Effects of Exposure to Environmental Tobacco Smoke
Topic: Smoking, Reproductive Health and Breast Cancer	
1983	Evidence that Heavy Smoking May Lower Age of Menopause
1994	Evidence that Women who Had Abortions Have Higher Risk of Breast Cancer
Topic: Prevalence of Breast Cancer	
1986	Lung cancer has surpassed breast cancer death in females
Topic: Aspirin Use and Breast Cancer	
1994	Significant Negative Relationship Between Aspirin Use and Breast Cancer
Topic: Dietary Explanations for Breast Cancer	
1997–1998	Carcinogens in Foods
Archive: Chemical Industry Documents and Fossil Fuel Industry Documents	
Topic: Glyphosate and Breast Cancer	
2013	Thongprakaisang et al. 2013 Study (Food and Chemical Toxicology): Animal study evidencing possible estrogen effects of glyphosate exposure.
2014	Séralini 2014 Mouse Study (Food and Chemical Toxicology): Long-term (2 year) exposure of rodents to Roundup (a glyphosate-inclusive compound) is found to be significantly related to the development of mammary tumors.
2015	International Agency for Research on Cancer 2015 statement on glyphosate carcinogenicity.
2015, 2017	Environmental Protection Agency (EPA), Cancer Assessment Review Committee 2015, 2017 review of glyphosate toxicity.
Topic: Benzene and Breast Cancer	
1970s	Occupational Safety and Health Administration 1970s review and designation of benzene as carcinogenic.
1980s	1980s Occupational Health Movement with antecedents in the 1930s–1950s.
1984	California Department of Health Services 1984 election of benzene “as a candidate substance for listing as a toxic air contaminant.”
Archive: Drug Industry Documents	
Topic: Premarin and Breast Cancer	
2002	The controversial 2002 findings of the Women’s Health Initiative (WHI) and Heart and Estrogen/Progestin Replacement Study Follow-Up (HERS II) cohort studies indicating the potential link between hormone replacement therapy and the development of breast cancer and heart disease.
Archive: Food Industry Documents	
Topic: Chemicals/Pesticides in Foods and Breast Cancer	
1969	Pesticides and Breast Cancer: Dept. of Health, Education and Welfare Commission on Pesticides and their Relationship to Environmental Health
1969	DDT and Breast Cancer: Cyclamate Ban under Delaney Clause could justify DDT ban
1971	Launch of War on Cancer—attention to environmental contaminants—National Cancer Act
1974	National Cancer Act (Amendment)
1978	Chemical and Physical Agents in the Environment Cause Cancer: Publication of The Politics of Cancer, Samuel Epstein, MD—vast media coverage and attention in Washington DC
1978	Publication of ‘Origins of Human Cancer’
1980s–2000s	Media attention to environmental/chemical causes of cancer (e.g., Love Canal, Alar Scare)
1993/5	National Academy of Sciences Report Pesticides in the Diet of Infants and Children (Philip Landrigan, Chair)
1996	Book—Our Stolen Future links DDT/DDE to breast cancer
1990s	Emerging Evidence on Endocrine Disruptors and breast cancer
Topic: Dietary Fat and Breast Cancer	
1975	Dietary Fat and Breast Cancer: Conference on Nutrition in the Causation of Cancer
1975	Dietary Fat and Breast Cancer: NCI forms Diet, Nutrition and Cancer Program Advisory Committee
1976	Burkitt’s Fiber Hypothesis of Cancer gaining popularity
1977	Dietary fat and breast cancer: US dietary goals
1978	Dietary Fat and Breast Cancer: NRC Diet, Nutrition and Cancer publishes interim guidelines
1979	National Cancer Institute Statement on Diet, Nutrition, and Cancer by Arthur Upton to Subcommittee on Nutrition, Senate Committee on Agriculture, Nutrition and Forestry
1980	NCI Statement on Diet, Nutrition, and Cancer by Arthur Upton to Subcommittee on Nutrition, Senate Committee on Agriculture, Nutrition and Forestry
1982	National Research Council panel concludes dietary fat suggestive of causal link to breast cancer
1983	NIH/NCI Prevention Subcommittee: Planning for nutrition and breast cancer studies
1985	NCI Cancer/breast cancer and nutrition studies—controversy over methodology
1990s	American Cancer Society names lung cancer as #1 cancer in women
Topic: Alcohol and Breast Cancer	
1977	Beer-drinking and breast cancer link: emerging research
1999	evidence of association between alcohol consumption and breast cancer
Topic: Synthetic Estrogen and Breast Cancer	
1977/78	Diethylstilbestrol in meat/dairy linked to breast cancer: emerging research
1978	DES in meat and dairy linked to breast cancer: Nader Group demands ban on DES due to breast cancer threat
Topic: Growth Hormones and Breast Cancer	
1995	Massachusetts introduces bill to require bovine somatotropin (BST) labeling for milk
1997	NYC proposes to ban purchase of rBST milk by city agencies and board of education (pushed by New York Green Party)
Topic: Obesity, Physical Activity and Breast Cancer	
2000s	Sugar/Obesity/Fiber links to cancer

^1^ These external events were identified by the research team during stage 1 as potential threats or opportunities (e.g., policymaking, emerging evidence in scientific or news/popular media) to the tobacco, chemical, fossil fuel, drug, and food industries based on preliminary searches of the UCSF IDL using search term “breast cancer”.

**Table 3:** Document Search Strategy and Number of Documents Returned During Initial and Focused Searches of the UCSF IDL by Topic.

Research Team Member	Archives/Collections Assigned	Documents Returned with Search Term “Breast Cancer”	Topics Selected by Project Team for In-Depth Review	Documents Returned with Additional/Focused Document Searches
Research Team Member 1	Truth Tobacco Industry Documents Archive Tobacco Institute	13,770	(1) Industry sponsored research (1970s–1980s) (2) Industry sponsored research (1990s)	“breast cancer AND grant” (CTR only) = 3231 “estrogen receptor” AND smoking = 241 “endocrine disruptor” = 20 “breast cancer advisory center” = 30 “breast cancer AND EPA” = 372 “hereditary AND breast cancer” = 1528 “breast cancer AND ETS” = 7794
	Center for Tobacco Research (CTR)	7377		
Research Team Member 2	Philip Morris	13,317	(3) 1997 California Environmental Protection Agency Reports on Environmental Tobacco Smoke (4) 2005 California Environmental Protection Agency Reports on Environmental Tobacco Smoke	“breast cancer” AND (“environmental tobacco smoke” OR “secondhand smoke” OR CALEPA OR “surgeon general” or “premenopausal” or “postmenopausal”) = 1067
	RJ Reynolds	8831		
	Lorillard	2615		
	Brown and Williamson	1365		
	American Tobacco	887		
	Topical Collections	2046		
	Additional Tobacco Documents	4075		
Research Team Member 3	Drug Industry Documents Archive	549	(5) Premarin	“breast cancer” and Premarin = 175 “women’s health research institute” = 79
	Chemical Industry Documents Archive	169	(6) Glyphosate	“breast cancer” AND glyphosate = 80 “GMO Answers” (Entire IDL) = 191
	Fossil Fuel Industry Documents Archive	15	(7) Benzene	“breast cancer” AND benzene (chemical and fossil fuel) = 64 documents “Environ Corporation AND benzene” (Entire IDL) = 212 “Exxon AND breast cancer” (Entire IDL = 938 documents
Research Team Member 4	Food Industry Documents Archive	842	(8) DDT residues in foods	“breast cancer” and (DDT OR DDE) = 102 “breast cancer” and (DDT or DDE) in entire IDL = 4119 “breast cancer” AND (“American Council on Science and Health” OR ASCH OR “international life sciences institute” OR ILSI) in entire IDL = 1781 “Harvard Human Nutrition Institute” = 221
			(9) Recombinant bovine somatotropin residue in milk	“Monsanto” (Stare collection) = 143
			(10) Dietary Fat	“fat AND breast cancer” = 626 “dietary fat and cancer” in entire IDL = 10,010

## Data Availability

Data supporting this manuscript are publicly available at https://www.industrydocuments.ucsf.edu/home/ (accessed between August 2021 and July 2022).

## Abbreviations

The following abbreviations are used in this manuscript:

CDOH	Commercial Determinants of Health
CBCRP	California Breast Cancer Research Program
IDL	Industry Documents Library
RFP	Request for Proposals
BCAction	Breast Cancer Action
CPAR	Critical Participatory Action Research
CE Studio	Community Engagement Studio

## References

[1] Green J., Thorogood N. (2018). Qualitative Methods for Health Research.

[2] Tasker K., Taketa R., Macquarie C., Deardorff A., Mani N.S., Cawley M.A. (2022). Digital Archives and Data Science: Building Programs and Partnerships for Health Sciences Research. Advances in Library and Information Science.

[3] UCSF Library (2023). Industry Documents Library/Bibliography—Publications Based on Industry Documents. Industry Documents Library.

[4] Bero L. (2003). Implications of the Tobacco Industry Documents for Public Health and Policy. Annu. Rev. Public Health.

[5] Hirschhorn N. (2005). The Tobacco Industry Documents: What They Are, What They Tell Us, And How to Search Them.

[6] Mamudu H.M., Gonzalez M., Glantz S. (2011). The Nature, Scope, and Development of the Global Tobacco Control Epistemic Community. Am. J. Public Health.

[7] WHO Framework Convention on Tobacco Control, World Health Organization (2003). WHO Framework Convention on Tobacco Control.

[8] UCSF Library (2025). Industry Documents Library/About IDL/Overview. Industry Documents Library.

[9] Anderson S.J., McCandless P.M., Klausner K., Taketa R., Yerger V.B. (2011). Tobacco documents research methodology. Tob. Control.

[10] Carter S.M., Little M. (2007). Justifying Knowledge, Justifying Method, Taking Action: Epistemologies, Methodologies, and Methods in Qualitative Research. Qual. Health Res..

[11] Balbach E.D. (2002). Tobacco industry documents: Comparing the Minnesota Depository and internet access. Tob. Control.

[12] Balbach E.D. (2005). Beyond quagmires: The evolving quality of documents research. Tob. Control.

[13] Malone R.E., Balbach E.D. (2000). Tobacco industry documents: Treasure trove or quagmire?. Tob. Control.

[14] Carter S.M. (2005). Tobacco document research reporting. Tob. Control.

[15] Collin J. (2012). Tobacco control, global health policy and development: Towards policy coherence in global governance. Tob. Control.

[16] California Breast Cancer Research Program (2021). Request for Proposal: Investigating Industry Influence Over Scientific Information on Breast Cancer and the Environment, Exploring the UCSF Industry Documents Library. https://cbcrp.org/files/pbc-funding/investigating-industry-influence-rfp.pdf.

[17] Breast Cancer Action (2024). Homepage. https://www.bcaction.org/.

[18] Clarke A.E., Friese C., Washburn R.S. (2018). Situational Analysis: Grounded Theory After the Interpretive Turn.

[19] Han E., Crosbie E., Ling P., Perez S., Khan H., Hiatt R., Kearns C. (2025). Tobacco industry influence on breast cancer research, policy and public opinion: Scoping the Truth Tobacco Industry Documents. Tob. Control.

[20] Kincheloe J.L. (2005). Critical Constructivism Primer.

[21] Kincheloe J.L. (2005). From Constructivism to Critical Constructivism. Critical Constructivism Primer.

[22] Fine M., Torre M.E. (2021). Essentials of Critical Participatory Action Research.

[23] Clarke A.E. (2005). Grounded Theory after the Postmodern Turn.

[24] Garrety K., Ward J.W., Warren C. (2006). Dietary Policy, Controversy, and Proof: Doing Something versus Waiting for the Definitive Evidence. Silent Victories.

[25] Hadorn G.H., Hoffmann-Riem H., Biber-Klemm S., Grossenbacher-Mansuy W., Joye D., Pohl C., Wiesmann U., Zemp E. (2008). Handbook of Transdisciplinary Research.

[26] Clarke A.E., Friese C., Washburn R.S. (2018). Doing Social Worlds/Arenas Maps. Situational Analysis: Grounded Theory After the Interpretive Turn.

[27] Joosten Y.A., Israel T.L., Williams N.A., Boone L.R., Schlundt D.G., Mouton C.P., Dittus R.S., Bernard G.R., Wilkins C.H. (2015). Community Engagement Studios: A Structured Approach to Obtaining Meaningful Input from Stakeholders to Inform Research. Acad. Med..

[28] Meharry-Vanderbilt Community Engaged Research Core (2009). Community Engagement Studio Toolkit 2.0. Vanderbilt Institute for Clinical and Translational Research. https://victr.vumc.org/wp-content/uploads/2019/07/CESToolkit-2.0.pdf.

[29] Fisher C.D. (2017). Padlet: An Online Tool for Learner Engagement and Collaboration, Available at https://Padlet.com. Acad. Manag. Learn. Educ..

[30] Clarke A.E., Friese C., Washburn R.S. (2018). Doing Positional Maps. Situational Analysis: Grounded Theory after the Interpretive Turn.

[31] Google (2024). Whiteboard Tools Reference & Glossary-Jamboard Help. https://support.google.com/jamboard/answer/7383648?hl=en.

[32] Maani N., Petticrew M., Galea S. (2023). The Commercial Determinants of Health.

[33] Pink S., Horst H., Lewis T., Hjorth L., Postill J. (2015). Digital Ethnography: Principles and Practice.

[34] Markham A.N., Denzin N.K., Lincoln Y.S. (2017). Ethnography in the Digital Internet Era: From Fields to Flows. The Sage Handbook of Qualitative Research.

